# Regional and global impacts of the 2007 Port-of-Spain Declaration on noncommunicable diseases

**DOI:** 10.26633/RPSP.2018.194

**Published:** 2019-03-29

**Authors:** John Kirton, W. Andy Knight, C. James Hospedales, Dinah Hippolyte, Julia Kulik

**Affiliations:** 1 University of Toronto University of Toronto TorontoOntario Canada University of Toronto, Toronto, Ontario, Canada.; 2 University of Alberta University of Alberta EdmontonAlberta Canada University of Alberta, Edmonton, Alberta, Canada.; 3 Caribbean Public Health Agency Caribbean Public Health Agency Port-of-Spain Trinidad and Tobago Caribbean Public Health Agency, Port-of-Spain, Trinidad and Tobago.; 4 University of the West Indies University of the West Indies St. Augustine CampusSt. Augustine Trinidad and Tobago University of the West Indies, St. Augustine Campus, St. Augustine, Trinidad and Tobago.

**Keywords:** Noncommunicable diseases, United Nations, World Health Organization, Pan American Health Organization, Caribbean Public Health Agency, West Indies, Enfermedades no transmisibles, Naciones Unidas, Organización Mundial de la Salud, Organización Panamericana de la Salud, Caribbean Public Health Agency, Indias Occidentales, Doenças não transmissíveis, Nações Unidas, Organização Mundial da Saúde, Organização Pan-Americana da Saúde, Caribbean Public Health Agency, Índias Ocidentais

## Abstract

**Objective.:**

To assess how well Caribbean regional institutions (RIs) met their commitments from the 2007 Port-of-Spain Summit (POSS) declaration on noncommunicable diseases (NCDs), and evaluate the POSS impact on the United Nations High-level Meeting (HLM) on NCDs in 2011 (2011 HLM), HLM NCD review in 2014 (2014 HLM), World Health Organization’s 2025 NCD targets (2025 WHO), and 2030 Sustainable Development Goals (SDGs) agreed upon in 2015.

**Methods.:**

This study uses a method developed by the University of Toronto’s Global Governance Program to measure institutions’ compliance with commitments from a summit and the match with commitments from earlier summits. This approach was supplemented using data from published literature, primary documents, and semistructured key informant interviews to detail how and why Caribbean RIs met the 2007 POSS commitments, how the 2007 POSS commitments led to compliance, and how the 2007 POSS influenced international NCD commitments.

**Results.:**

Caribbean RIs implemented the 2007 POSS commitments better when they had more public legitimacy, when their missions aligned with those commitments, and when more resources were available to them. Implementation constraints arose from multiple, sometimes competing, interests of the decision-making and national implementing bodies of the Caribbean Community (CARICOM). Internationally, the early, expanding efforts of the POSS pioneers had an initially important but subsequently diminishing impact on the HLMs.

**Conclusions.:**

For the Caribbean region, the Caribbean Public Health Agency should be funded to lead strengthened Caribbean RIs in coordinated action on NCDs. At the international level, the United Nations should embed NCDs in a “whole-of-global-governance” approach, monitor implementation annually, foster transregional partnerships on NCD-related themes, engage civil society, and support regular regional and global summits to enhance implementation and improvement, aimed at future HLMs on NCDs, the 2025 WHO targets, and the SDG NCD targets.

## RESUMEN

**Objetivo.:**

Evaluar el grado de cumplimiento de las instituciones regionales del Caribe con los compromisos adquiridos en la Declaración de la Cumbre de Puerto de España de 2007 sobre las enfermedades no transmisibles (ENT), y evaluar el impacto de esta Cumbre en la Reunión de Alto Nivel de las Naciones Unidas sobre las ENT de 2011, la Reunión de Revisión de 2014, las metas sobre ENT para 2025 de la Organización Mundial de la Salud (OMS), y los Objetivos de Desarrollo Sostenible 2030, acordados en 2015.

**Métodos.:**

Este estudio utilizó un método desarrollado por el Programa de Gobernanza Global de la Universidad de Toronto para medir el cumplimiento de las instituciones con los compromisos de una cumbre y la compatibilidad con los compromisos de cumbres anteriores. Este enfoque se complementó con datos de la bibliografía, documentos primarios y entrevistas semiestructuradas con informantes clave para detallar cómo y por qué las instituciones regionales del Caribe cumplieron con los compromisos de la Cumbre de Puerto de España de 2007, cómo los compromisos de esta Cumbre llevaron a su cumplimiento, y cómo la Cumbre influyó en los compromisos internacionales relacionados con las ENT.

**Resultados.:**

Las instituciones regionales del Caribe implementaron mejor los compromisos de la Cumbre de Puerto de España de 2007 cuando disponían de mayor legitimidad pública, cuando sus misiones se alineaban con esos compromisos y cuando disponían de más recursos para afrontarlos. Las restricciones en la implementación se relacionaron con la existencia de intereses múltiples –y a veces contrapuestos– de los organismos tomadores de decisiones y los organismos responsables de la implementación a nivel nacional en la Comunidad del Caribe (CARICOM). A nivel internacional, los primeros esfuerzos de expansión de los pioneros de la Cumbre de Puerto de España tuvieron un impacto importante al inicio de las reuniones de alto nivel, pero posteriormente este disminuyó.

**Conclusiones.:**

En la región del Caribe, la Agencia de Salud Pública del Caribe (CARPHA) debe disponer de financiación para liderar a las instituciones regionales del Caribe en una acción coordinada contra las ENT. A nivel internacional, las Naciones Unidas deben integrar las ENT en un enfoque de “gobernanza global”, monitorear la implementación de manera anual, fomentar asociaciones transregionales en temas relacionados con las ENT, involucrar a la sociedad civil y apoyar la realización regular de cumbres regionales y mundiales para mejorar su implementación y mejora, con miras a futuras reuniones de alto nivel sobre ENT, las metas de la OMS 2025 y las metas para las ENT de los Objetivos de Desarrollo Sostenible 2030.

## RESUMO

**Objetivo.:**

Avaliar o grau de cumprimento das instituições regionais do Caribe com os compromissos assumidos na Declaração do Cume de Porto da Espanha de 2007 sobre as doenças não transmissíveis (DNT), e avaliar o impacto deste Cume sobre a Reunião de Alto Nível das Nações Unidas sobre as DNT de 2011, a Reunião de Revisão de 2014, as metas sobre DNT para 2025 da Organização Mundial da Saúde (OMS) e os Objetivos de Desenvolvimento Sustentável 2030, concordados em 2015.

**Métodos.:**

Este estudo utilizou um método desenvolvido pelo Programa de Governança Global da Universidade de Toronto para medir o cumprimento das instituições com os compromissos de um cume e a compatibilidade com os compromissos de cumes anteriores. Este enfoque foi complementado com dados da bibliografia, documentos primários e entrevistas semi-estruturadas com informantes chave para detalhar como e por que as instituições regionais do Caribe cumpriram os compromissos do Cume de Porto da Espanha de 2007, como os compromissos deste Cume levaram ao seu cumprimento, e como o Cume influiu nos compromissos internacionais relacionados com as DNT.

**Resultados.:**

As instituições regionais do Caribe implementaram melhor os compromissos do Cume de Porto da Espanha de 2007 quando dispuserem de maior legitimidade pública, quando suas missões se alinhavam com esses compromissos e quando dispunham de mais recursos para confrontá-los. As restrições na implementação se relacionaram com à existência de interesses múltiplos–e às vezes contrapostos–dos organismos responsáveis pela tomada de decisão e or organismos responsáveis pela implementação no nível nacional na Comunidade do Caribe (CARICOM). No plano internacional, os primeiros esforços de expansão dos pioneiros do Cume de Porto da Espanha tiveram um impacto importante no início das reuniões de alto nível, mas posteriormente este diminuiu.

**Conclusões.:**

Na região do Caribe, a Agência de Saúde Pública do Caribe (CARPHA) deve dispor de financiamento para liderar as instituições regionais do Caribe em uma ação coordenada contra as DNT. No plano internacional, as Nações Unidas devem integrar as DNT em um enfoque de “governança global”, monitorar a implementação de maneira anual, promover associações transregionais em temas relacionados com as DNT, envolver a sociedade civil, e apoiar a realização regular de cumes regionais e mundiais para melhorar sua implementação e melhora com vistas a reuniões de alto nível futuras sobre DNT, as metas da OMS 2025 e as metas para as DNT dos Objetivos de Desenvolvimento Sustentável 2030.

In 2007, the world’s first summit dedicated to noncommunicable diseases (NCDs) was held by the heads of government of the Caribbean Community (CARICOM) in Port-of-Spain, Trinidad and Tobago. At that assembly, the landmark Declaration of Port-of-Spain: Uniting to Stop the Epidemic of Chronic NCDs was issued. The Declaration contained 27 commitments for NCD prevention and control, including reducing risk factors and improving access to preventive care (Table 1). Additionally, it entrusted to governments, civil society, and the private sector (“all of society”), working together, regular monitoring and evaluation of commitments, and it assigned specific responsibilities to Caribbean regional institutions (RIs) for meeting those commitments (Table 2).

**TABLE 1:** Matching commitments of the 2007 Port-of-Spain summit (POSS) Declaration and of the United Nations 2011 High-level Meeting (2011 HLM) of the General Assembly on the Prevention and Control of Non-communicable Diseases (NCDs)

2007 POSS commitment^a^	Average compliance^b^	Matching 2011 HLM commitments^c^	Frequency of match between POSS and 2011 HLM commitments	Strength of match between POSS and 2011 HLM commitments
POSS-01: Strengthen regional institutions	−0.25	91, 92, 93, 94, 95, 96, 97, 98, 99, 100, 101, 102, 103, 104, 105, 106, 107, 108, 109, 123, 124, 125, 134	23	1
POSS-02: Legislate the Framework Convention on Tobacco Control^d^	−0.07	1, 17, 33, 68, 163	5	2
POSS-03: Ban smoking in public places	−0.50	0	0	0
POSS-04: Ban tobacco sales to children	−0.64	0	0	0
POSS-05: Ban tobacco advertising to children	−0.50	0	0	0
POSS-06: Ban promoting tobacco to children	−0.57	0	0	0
POSS-07: Require warning labels for tobacco	−0.62	5, 21, 37	3	1
POSS-08: Implement fiscal measures against tobacco	+0.14	13, 25, 29, 41, 45	5	2
POSS-09: Obtain revenue from tobacco and alcohol	−0.14	0	0	0
POSS-10: Implement screening programs	−0.45	86, 133, 145, 146, 147, 148, 149, 150, 151, 162	10	2
POSS-11: Mandate physical education in schools	+0.65	0	0	0
POSS-12: Provide incentives/resources for physical education in schools	+0.40	0	0	0
POSS-13: Promote healthy meals/eating through the education sector	−0.10	2, 6, 22, 38, 80, 81	6	2
POSS-14: Promote food security^d^	−0.25	0	0	0
POSS-15: Eliminate trans fats	−0.30	75, 79	2	3
POSS-16: Promote fair trade	−0.15	82, 157	2	2
POSS-17: Require food labeling for nutrition	−0.20	0	0	0
POSS-18: Promote mass physical education	−0.70	3, 7, 11, 15, 19, 23, 27, 31, 35, 39, 43, 47, 49, 50, 51, 52, 53, 54, 55, 56, 57, 58, 59, 60, 61, 62, 63, 64, 65, 66, 67	31	3
POSS-19: Develop parks for physical education	−0.65	69	1	3
POSS-20: Use gender-based approaches	−0.90	115, 116	2	3
POSS-21: Offer incentives for public education on wellness	+0.10	87, 88, 89, 90, 193, 194	6	3
POSS-22: Offer incentives for public education on changing behavior	0	195, 197	2	3
POSS-23: Incentives for public education on NCD self-management	−0.45	0	0	0
POSS-24: Engage media partners	0	180	1	1
POSS-25: Conduct research surveillance	+0.25	131, 132, 189, 190, 191, 192, 196, 198, 199, 200, 201, 202, 203, 204, 205	15	2
POSS-26: Perform monitoring and evaluation	0	113, 188	2	1
POSS-27: Promote Caribbean Wellness Day	+0.35	0	0	0

***Source:*** Prepared by the authors, based on the study results.

a For the full text of the commitments see Charting Compliance with the 2007 Port of Spain summit Commitments on Non-communicable Diseases at http://www.ghdp.utoronto.ca/compliance/commitments.shtml.

b Each member state of the Caribbean Community was assessed by the authors for compliance with the commitments expressed in the POSS Declaration within a year of the 2007 summit and combined here in an average score per commitment. Compliance was measured using a three-point scale where +1 indicates full compliance, 0 indicates partial compliance or a work in progress, and −1 indicates no compliance or actions taken antithetical to the commitment.

c Numbers correspond to the list of 205 commitments identified by the authors in the outcome document of the United Nations High-level Meeting of the General Assembly on the Prevention and Control of Non-communicable Diseases in 2011, which are available upon request.

d Compliance with these commitments was also monitored for the second, third, fourth, and fifth year after the 2007 POSS, with the following results: POSS-02 compliance: Year 2 (2009) +0.10; Year 3 (2010) +0.20; Year 4 (2011) +0.20; Year 5 (2012) +0.45; POSS-14 compliance: Year 2 (2009) +0.35; Year 3 (2010) +0.35; Year 4 (2011) +0.35.

Existing studies have analyzed the preparations, production, and outcomes of the Port-of-Spain Summit (POSS) and their impacts in the short term (1-4). However, none of those studies considered the longer-term impacts of the POSS in terms of the role of Caribbean RIs in helping CARICOM members comply with their commitments and how the POSS and the CARICOM members’ compliance contributed to the creation, outcomes, and impacts of the global United Nations high-level meetings (HLMs) focused on NCDs in the following eight years. This article takes up these tasks.

This article asks what the longer-term regional and global impacts of the POSS were, as the world’s first summit on preventing and controlling NCDs. It answers by assessing CARICOM members’ compliance with their POSS commitments, how Caribbean RIs met their summit commitments, and how the POSS influenced the United Nations High-level Meeting of the General Assembly on the Prevention and Control of Non-communicable Diseases on 19–20 September 2011 (2011 HLM), the high-level meeting of the General Assembly on the prevention and control of NCDs on 10 July 2014 (2014 HLM), and the United Nations 2030 Sustainable Development Goals (SDGs), particularly SDG3 (on health) agreed upon at the United Nations Sustainable Development Summit (SDG Summit) on 25–27 September 2015.

**TABLE 2:** Regional institutions assigned mandates under the 2007 Port-of-Spain Declaration on noncommunicable diseases

Category	Regional institution(s)
Pan American Health Organization	Caribbean Food and Nutrition Institute Caribbean Epidemiology Centre
Caribbean Community (CARICOM)	CARICOM Cooperation in Health Secretariat Office of Trade Negotiation (previously Caribbean Regional Negotiating Machinery) Caribbean Regional Organization for Standards and Quality Caribbean Public Health Agency (operational since 2013) Caribbean Agricultural Research and Development Institute
Associate	The University of the West Indies
Regional civil society	Healthy Caribbean Coalition (established 2008)

***Source:*** Prepared by the authors, based on the study results.

## METHODS

This study uses a method developed by the University of Toronto’s Global Governance Program to measure members’ compliance with commitments from a particular summit and how well those commitments matched commitments from a previous summit (5-9).

This analysis employs a framework first developed by George von Furstenberg and Joseph Daniels for assessing compliance with Group of Seven (G7) summit objectives, supplemented by Marina Larionova’s assessment of G7 members’ compliance with international and regional organizations’ mandates (10-11). For each commitment in this analysis, each institution’s country member received a score of +1 for full compliance, 0 for partial compliance or a work in progress, or −1 for no compliance or actions taken antithetical to the commitment, during the year after the commitment was made.

Using data collected from published literature, primary documents, and semistructured key informant interviews, the analysis also identifies the influence of the POSS on NCD-related actions taken by regional and global organizations.

Relevant published literature was identified through scanning peer-reviewed journals on public health and on global governance. These journals included the *Bulletin of the World Health Organization*, *Revista Panamericana de Salud Pública/Pan American Journal of Public Health*, *Lancet, British Medical Journal, Global Heart, American Journal of Public Health, Health and Place, World Medical and Health Policy,* *Global Governance, International Organization, International Affairs*, and *Administrative Sciences.* This was supplemented by a systematic search using the University of Toronto Libraries database for combinations of key words on noncommunicable diseases, heart and stroke, cancer, diabetes, chronic respiratory disease, Caribbean Community, summit, United Nations high-level meeting, and Sustainable Development Goals. The most relevant articles are identified in the works cited and in the references of this article.

Primary documents included those in the global summitry archives of the Global Governance Program at the University of Toronto and those provided by the Caribbean Public Health Agency (CARPHA) and key informants. A total of 50 semistructured interviews were conducted between 2010 and 2017, with current and former officials of the institutions of interest. RI responses were assessed according to raw information provided by these key informants, triangulated with and confirmed by publicly available data on specific implementing actions and, in some cases, progress reports provided by the Caribbean RIs themselves.

Following standard practice, RIs were conceived as both “actors” and “frameworks for action” (12). They provided policy-making frameworks that imbued them with consultative power. Caribbean states usually vest decision-making power in bodies of state representatives and reserve implementation/enforcement power for the national level. Caribbean RIs typically support national implementation through training and technical advisory services, research and development, collection and critical analyses of regional data to guide policy development, monitoring and evaluation, sharing of lessons learned, networks and partnerships, and the mobilization of resources to implement policies and programs.

More specifically, CARICOM RIs’ responses were assessed according to those RIs’ speed of starting to implement each mandate, the continuity and completeness of that implementation, and the extent to which members of RIs complied with related commitments.

This POSS influence was assessed in a three-step process. First, the POSS Declaration was matched with the 2011 HLM outcome document. Second, the 2011 HLM outcome document was matched with the outcome document from the 2014 HLM. Third, members’ compliance with the commitments contained in all the outcome documents was measured, using methods developed by the University of Toronto’s Global Governance Program. The assessment next traced, through key informant interviews using a snowball technique, the political and diplomatic process from the POSS to the SDG Summit in 2015, at which the SDG targets were agreed upon, using the same methods as those used for the Caribbean RIs, thus adding to works based on research done before the 2011 HLM (13-17).

This research was conducted for the Port-of-Spain Declaration Evaluation Research Group (POSDeval) led by the University of the West Indies (UWI), Cave Hill campus, and funded by Canada’s International Development Research Centre on behalf of the Pan American Health Organization (PAHO) and CARICOM (11).

## RESULTS

### Assessing regional institutions

The POSS Declaration issued 27 commitments that reflected the cognizance of the CARICOM heads of government of their governments’ capacity constraints and the heads’ resolve to have Caribbean RIs provide collective expertise and technical resources to facilitate compliance with those commitments.

Very few studies have analyzed either Caribbean RIs’ responsiveness to the POSS commitments or the impact of these responses on CARICOM members’ compliance with those commitments. The Healthy Caribbean Coalition (HCC), a not-for-profit civil society organization comprising over 100 nongovernmental organizations committed to preventing and combatting NCDs in the Caribbean, was one of the few bodies to assess Caribbean RIs’ responses to their POSS commitments, but it did not address factors influencing responsiveness (19). POSDeval provided a baseline for monitoring the responses of Caribbean RIs and their support of member-state compliance. It identified nine Caribbean RIs that were assigned 13 commitments by the POSS Declaration. In some cases, the same commitment was issued to several RIs, which are grouped in Table 2.

The speed of responses and completeness of implementing activities varied (data available upon request). The Caribbean Food and Nutrition Institute (CFNI), the Caribbean Epidemiology Centre (CAREC), CARPHA, the Office of Trade Negotiation (OTN), the Caribbean Agricultural Research and Development Institute (CARDI), UWI, and the HCC were timely in their responses and addressed the full range of activities required to fulfill their mandates throughout the evaluation period. (CFNI and CAREC merged into CARPHA in 2013.) The Caribbean Cooperation in Health (CCH) Secretariat and the Caribbean Regional Organisation for Standards and Quality (CROSQ) fared well in their breadth and continuity; however, there was a slight lag in responding to the POSS commitments.

Caribbean RI responses related to member compliance with the POSS Declaration varied significantly among the institutions and their specific institutional mandates (data available upon request). Member compliance with commitments related to the mandates of PAHO institutions, UWI, and CCH was much higher than compliance with commitments related to CROSQ and OTN; compliance requiring collaboration with these two CARICOM institutions was significantly lower.

Three reinforcing factors supported the speed and ability of Caribbean RIs to undertake activities to meet their POSS mandates and related commitments: institutional legitimacy, material resources, and alignment of the institutions’ mandates with the commitments. The interests of the decision-making bodies and implementing agencies were critical to Caribbean RIs’ impact on member compliance. When the mandates aligned with those interests, Caribbean member states were more likely to adopt the RIs’ policy recommendations. CFNI was wound down in 2012, before CARPHA became operational, which perhaps affected CARPHA’s responsiveness to the POSS diet and nutrition commitments.

The POSS Declaration reinforced the institutional legitimacy of existing Caribbean RIs and ascribed legitimacy to new regional institutions (12). This legitimacy facilitated institutions’ engagement with stakeholders and bolstered support for their programs and activities. The mandate issued by the Declaration to civil society to prevent and control increased cases of NCDs was critical in bolstering the HCC’s success in engaging stakeholders. In 2013, the HCC was selected as the regional implementing partner for the NCD Alliance, which brings together more than 2 000 civil society organizations in over 170 countries (20). In 2014, the HCC produced the Caribbean’s first status report on the region’s civil society responses to NCDs (19).

Similarly, the mandate issued to OTN to pursue fair trade policies legitimized its advocacy for prioritizing health in negotiating multilateral trade agreements and assessing the implications of global trade policies on controlling and preventing obesity.

Caribbean RIs struggled to address the POSS Declaration commitments using existing institutional and budgetary resources. The POSS did not authorize new monies to fund the mandates or create implementation mechanisms. Subsequent strategies neither assigned nor identified additional funding for these institutions. The ability to utilize existing resources or access additional funding was crucial. CAREC and CFNI were financed through PAHO’s regional budget allocations and extrabudgetary projects. OTN could use existing in-house technical resources, and the HCC successfully attracted regional funding (21) and international grants funding (22) to support its advocacy and monitoring efforts. (The HCC collaborated with the Commonwealth Secretariat to launch the NCD Commissions Strengthening Project to enhance multisectoral coordination and foster action by deepening engagement with the regional private sector (20)). CARPHA partially bridged its resource gap by leveraging its institutional legitimacy to form strategic partnerships with traditional and nontraditional bodies that provided technical and financial resources. (CARPHA worked with, among others, the U.S. Centers for Disease Control and Prevention, International Agency for Research on Cancer, Public Health Agency of Canada, International Development Research Centre, World Diabetes Foundation, European Union, and International Development Law Organization.) With PAHO’s support, CARPHA engaged Argentina, Chile, and Mexico in South-South cooperation to transfer know-how and successful practices.

To leverage the overarching structure of CARICOM to support CARPHA’s mandate, CARPHA developed platforms that provided sustained involvement with relevant RIs (23). CARPHA engaged national implementing agencies and bodies in the private sector (24) to encourage buy-in from the actors responding to its mandate and to increase members’ compliance with the 2007 POSS commitments. Importantly, CARPHA engaged CARICOM’s Council of Ministers to encourage action on fair trade policies, develop standards for food imports aimed at reducing the obesogenic environment, and improve access to nutritious foods, thereby reducing the level of NCDs (25).

CARPHA also encouraged dialogue between regional decision-making bodies (26). In collaboration with the CCH Secretariat, it engaged a meeting of the Council for Trade and Economic Development (COTED) in 2015 on the issue of preventing childhood obesity in the Caribbean (27). The CARICOM Heads of Government Meeting in 2016 then mandated that the Council for Human and Social Development and COTED work jointly on the issue. In February 2017, a consortium of 12 regional economic and social sector institutions developed a road map to accelerate multisector action on CARPHA’s policy package for healthier food environments (28), supported by evidence briefs from multiple stakeholders (29).

When the assigned commitments were aligned with Caribbean RIs’ existing missions, the response speed was not hampered by the extra effort that often accompanies institutions with assigned mandates acting outside their core missions. The CFNI, CARDI, CAREC, CCH, and OTN were already engaged in activities that fell within the scope of their 2007 POSS Declaration mandates and related commitments. OTN was already exploring the intersection of health and trade and reviewing the use of trade policies to address NCDs. The CCH Secretariat had an established regional mechanism for coordinating stakeholder consultation and developing regional health frameworks and strategic plans.

### Assessing the global impact

POSS also had an early, significant influence on the global governance of NCDs, through the 2011 HLM, the 2014 HLM, and the 2015 SDG Summit, although that influence progressively diminished. The 2011 HLM, held only four years after the POSS, was the second-ever United Nations summit devoted to health (9). The POSS pioneers, leaders, and governments provided the essential initial global ambition, vision and strategy, continuous efforts, and expanding array of international allies to produce the global results.

The 2011 HLM outcome document had only one direct reference to the POSS, but the document put the reference in its preamble’s paragraph, which expressed United Nations appreciation for various regional initiatives to control and prevent NCDs. The 2011 HLM’s 205 precise, future-oriented, politically obligatory and binding commitments substantially matched the 27 POSS commitments (Table 1). A majority (56%) of the 2011 HLM commitments had at least one POSS precursor commitment (Table 1). The rest (44%) came from new subjects, such as alcohol use, accountability, and climate change (9).

Reciprocally, a majority (59%) of the POSS commitments were reflected in the 2011 HLM ones at least once (Table 1 shows the matches but no percentages). Eight POSS commitments matched five or more 2011 HLM ones, and seven highly matched at least one (Table 1). However, a minority (31%) of the 2011 HLM commitments matched those of the 2014 HLM (data available upon request).

United Nations members’ compliance with 2011 HLM commitments in the first year after that HLM shows an influential “POSS” push through to 2012 (data available upon request). Of the eight commitments initially assessed for compliance, the six with a matched POSS precursor commitment (i.e., parent) had higher compliance by the United Nations’ 40 Western Hemisphere members and by the CARICOM members specifically (data available upon request). If the POSS parent had high first-year compliance from CARICOM members, its matched 2011 HLM offspring had higher first-year compliance too (data available upon request).

More specifically, the six 2011 HLM commitments with a POSS parent had an average compliance of +0.27 by Western Hemisphere members, compared to −0.11 for the two commitments without a parent (data available upon request). The increase was even higher for the CARICOM members. However, there was also a similar increase among CARICOM members for commitments without a POSS match, suggesting a more general POSS participation effect.

Among the 13 2011 HLM commitments subsequently assessed for first-year compliance, the 9 commitments with POSS parents had average compliance +0.27 higher among CARICOM members than among the other United Nations members of the Western Hemisphere (data available upon request). For the remaining 4 commitments, the average compliance for CARICOM members was −0.07 lower than for the other Western Hemisphere members.

This POSS influence was enhanced by higher first-year compliance from CARICOM members with their parent POSS commitments (data available upon request). The three POSS commitments with the highest first-year compliance, averaging +0.14, had their three matched 2011 HLM offspring secure first-year compliance of +0.47. The three POSS commitments with the lowest compliance, averaging −0.49, had their three matched 2011 HLM offspring secure first-year compliance of only +0.09.

Assessing CARICOM members’ compliance with their 27 POSS commitments in annual intervals, as measured by the implementation of 14 indicators set out by the CCH in Phase III of the Regional Caribbean Cooperation Strategy, suggests a sustained positive POSS push (data available upon request). (The CCH III process refers to CARICOM’s Caribbean Cooperation in Health Phase III – Regional Health Framework (2010–2015) – “Investing in Health for Sustainable Development.”)

The political process through which the regional POSS shaped the United Nations’ global NCD regime began by producing POSS with a global vision. The first phase began in 1986, when CARICOM health ministers approved the CCH’s first initiative in which NCDs were a priority. The CARICOM heads of government then approved a regional approach to NCDs at their meeting in 2001 in Nassau, Bahamas. In July 2006 they approved “a special regional consultation” hosted by Trinidad and Tobago, and in February 2007 upgraded it to a regional “summit,” also held in Trinidad and Tobago. The POSS was held in Port-of-Spain in September 2007.

The initiative to produce POSS had begun with three members of the Caribbean Commission on Health and Development, some of whom sought a CARICOM summit as a pathway to a United Nations one (30). Essential financial support came from outside the Caribbean region, specifically from Canada. The 2007 POSS planners focused the agenda on prevention, leading to CARICOM commitments on physical activity, nutrition, healthy diets, smoking reduction, preventive treatment and education, surveillance, monitoring, and evaluation, but not on reducing alcohol use. Leaders also agreed to make the second Saturday every September “Caribbean Wellness Day,” and that led to the PAHO-designated “Wellness Week” (1, 31).

The second phase started right after POSS, when CARICOM heads of government were told that they needed to take their work further. With PAHO’s encouragement, they injected NCDs into their broader Commonwealth Heads of Government Meeting and the Summit of the Americas, both hosted in Port-of-Spain in 2009. Within the Americas, other regional blocs in Central and South America issued declarations on NCDs (specifically the Central American, Andean, and Southern Cone integration movements).

At the United Nations, key individuals from the Caribbean lobbied the United Nations secretary general to hold a high-level meeting on NCDs. They drafted the authorizing resolution in a way that aligned with POSS to benefit all regions.

In 2007, the World Health Organization (WHO) began crafting a global plan on NCDs. PAHO/WHO members soon recognized that preventing and controlling NCDs required a political solution involving the highest level of policymakers, namely heads of government. The Caribbean diplomatic missions in Geneva and New York, delegations from Caribbean countries attending WHO and United Nations meetings, and the Norwegian and Russian representatives pushed to pass resolutions at the annual World Health Assembly and at the United Nations, calling for a high-level meeting on NCDs in 2011. Each of the six WHO regions met in the lead-up to the HLM. The first Global Ministerial Conference on Healthy Lifestyles and Noncommunicable Disease Control, held in Moscow in May 2011, provided additional support.

During the negotiations on the 2011 HLM outcome document, disagreements arose over targets and indicators, financing, WHO or United Nations leadership, the use of WHO’s “best buys,” and the addition of mental health to the four major NCDs of cancer, diabetes, respiratory disease, and cardiovascular disease. Some United Nations members disagreed with using the term “best buys” but referenced the WHO text that listed those measures. Influential individuals from the Caribbean helped to focus the 2011 HLM on the big four NCDs, as POSS had done. Those individuals also shaped other outcomes in POSS-consistent ways.

In the third phase, creating the subsequent 2014 HLM, the direct Caribbean contribution diminished, even though Trinidad and Tobago led the Group of 77 developing countries at the United Nations (17). Although the 2014 HLM was pitched at the political level, no country leaders came.

The next United Nations HLM on NCDs occurred at the United Nations in New York on 27 September 2018. The declaration from that meeting was titled “Time to Deliver: Accelerating Our Response to Address NCDs for the Health and Well-Being of Present and Future Generations.” It emphasized the need to mobilize further political action at the national and global levels to prevent and control NCDs. The next United Nations HLM on NCDs is scheduled for 2025.

The fourth phase, securing the SDGs with NCDs included, relied even more on WHO (32, 33). WHO officials had long shaped their HLM preparations to include health and NCDs in the UN’s sustainable development agenda for the SDG Summit in 2015. After the 2011 HLM, they started to prepare road maps. In drafting their strategic plan and the global framework, which included WHO’s 2025 NCD targets, they remained focused on the SDGs. The facilitators of the 2011 United Nations General Assembly ensured that NCDs were incorporated in the SDG preparatory process.

The result was the 2015 SDG Summit’s inclusion of an NCD target (reduce premature deaths from NCDs 30% by 2030) in SDG3 on health, as well as the identification of health-supporting measures in other SDGs. The concepts of health throughout the human life cycle and moving from an illness framework to a wellness one were also advanced.

## DISCUSSION

This analysis aimed to assess how Caribbean regional institutions met their commitments from POSS, the first political summit on NCDs. The study found that public legitimacy, mission alignment with POSS, and resources enhanced RI compliance. Furthermore, when RI commitments aligned with national interests, CARICOM member-state compliance with the POSS commitments was much higher.

When Caribbean RIs’ overall mandates aligned with the interests of respective national decision-making bodies and implementing agencies, member-state compliance with the commitments in the POSS Declaration was much higher. In addition to two Caribbean RIs (CARPHA and the CCH Secretariat), two PAHO decision-making bodies (CFNI and CAREC) were composed of Caribbean health ministers. Similarly, the national implementing bodies of those RIs consisted of health officials who shared similar health interests. These actors played a key role in convening the 2007 POSS and were intimately aware of the directives issued in the POSS Declaration.

In contrast, the decision-making body and implementing agencies of OTN and CROSQ are composed of trade ministers and officials accountable to various stakeholders. The processes of those bodies were often stymied as officials sought to resolve competing interests. An example is the CROSQ’s response to its mandate to standardize effective tobacco warning labels. Controlling tobacco use is one of the commitments in the POSS Declaration. It is also a primary mandate of the CROSQ when it comes to standard-setting and quality control. During the CROSQ consultation process, Trinidad and Tobago tobacco manufacturers vigorously opposed initial schedules for rotating health warnings because they felt such warnings would increase their cost of doing business. Some national health-based organizations rejected the tobacco warning labels because they were not far-reaching enough.

This analysis highlights the value of funding CARPHA to lead strengthened Caribbean RIs to prevent and control the rising burdens of NCDs. The analysis further shows the value of embedding NCDs in the biggest, broadest, whole-of-global-governance approach at the United Nations and of engaging the private sector, civil society, and mass publics. It also suggests the value of annual compliance monitoring and regular summits to review, reinforce, and improve implementation, now aimed at the 2025 HLM on NCDs and the 2030 SDGs review conference.

With the global and regional burden of NCDs still rising rapidly, but with NCDs as only one of the 169 targets in the SDGs, new actions from and in the Caribbean are needed now. These could include regular CARICOM summit sessions on NCDs, interministerial council meetings to review and improve implementation, a streamlined monitoring mechanism benefiting all key actors, the addition of NCDs to the agenda of the European Union’s Latin America and the Caribbean meetings, and continuous comprehensive assessments of compliance with NCD-related commitments from the POSS, CARICOM heads of government meetings, HLMs, and the SDGs, with a focus on the fiscal, economic, and environmental benefits that compliance brings.

To show continued global leadership, the CARICOM Quasi Cabinet, the Council for Trade and Economic Development, and the Caribbean finance ministers and foreign ministers should use the intersessional conferences and heads of government meetings to take key decisions on smoke-free public spaces and mandatory food labeling as human rights and consumer empowerment issues. They should also adjust the public tax structure to reduce the consumption of tobacco, alcohol, and sugar-sweetened beverages by Caribbean citizens. To accelerate action, more research on implementation is needed, particularly focusing on barriers to the uptake of evidence, political feasibility, and the building of all-of-society alliances.

## Funding.

Research conducted for this analysis was funded by the International Development Research Centre.

## Disclaimer.

Authors hold sole responsibility for the views expressed in the manuscript, which may not necessarily reflect the opinion or policy of the *RPSP/PAJPH* or PAHO.
